# Oxidative balance score and risk of cancer: a systematic review and meta-analysis of observational studies

**DOI:** 10.1186/s12885-023-11657-w

**Published:** 2023-11-24

**Authors:** Motahareh Hasani, Seyedeh Parisa Alinia, Maryam Khazdouz, Sahar Sobhani, Parham Mardi, Hanieh-Sadat Ejtahed, Mostafa Qorbani

**Affiliations:** 1 Department of Nutrition, School of Public Health, Golestan University of Medical Sciences, Gorgan, Iran; 2 Student Research Committee , Alborz University of Medical Sciences, Karaj, Iran; 3 Growth and Development Research Center, Tehran University of Medical Sciences, Tehran, Iran; 4 Non-Communicable Diseases Research Center, Alborz University of Medical Sciences, Karaj, Iran; 5 Obesity and Eating Habits Research Center, Endocrinology and Metabolism Clinical Sciences Institute, Tehran University of Medical Sciences, Tehran, Iran; 6 Endocrinology and Metabolism Research Center, Endocrinology and Metabolism Clinical Sciences Institute, Tehran University of Medical Sciences, Tehran, Iran; 7 Chronic Diseases Research Center, Endocrinology and Metabolism Population Sciences Institute, Tehran University of Medical Sciences, Tehran, Iran

**Keywords:** Oxidative balance score, Cancer, Malignancy, Neoplasms

## Abstract

**Background:**

The oxidative balance score (OBS) has been utilized to assess the overall pro- and antioxidant exposure status in various chronic diseases. The current meta-analysis was carried out to pool the association between OBS and the risk of cancer.

**Methods:**

We systematically searched the Web of Science, PubMed, Scopus, Embase, and Google Scholar up to August 2023. All observational studies which evaluated the association of OBS with the risk of cancers were included. There was no time of publication or language restrictions. Heterogeneity between studies was assessed using the Chi-square-based Q-test and the I^2^. A random-effects model meta-analysis was conducted to estimate the pooled effect sizes. Possible sources of heterogeneity were explored by subgroup and meta-regression analysis.

**Results:**

Totally, 15 studies (9 case–control and 6 cohorts) were eligible for meta-analysis. Random effect model meta-analysis of case–control studies showed that higher OBS significantly decreases the odds of cancers (pooled OR: 0.64, 95% CI: 0.54, 0.74). In the cohort studies, the association of OBS with the risk of cancers was not significant (pooled HR: 0.97, 95% CI: 0.80,1.18). The subgroup analysis showed that cancer type and gender were the potential sources of heterogeneity.

**Conclusion:**

Our results show an inverse and significant association between higher OBS and odds of colorectal cancers in case–control and cohort studies. In the case of prostate cancer in cohort studies, our results did not align with the hypothesis. Considering the importance of diet and antioxidant balance in the conditions of malignancy, it is suggested to conduct more comprehensive studies with standard measurement methods to obtain conclusive results.

## Introduction

Cancer has emerged as a prominent global illness, posing a significant hazard to the well-being of humanity [1]. Annually, the American Cancer Society predicts the occurrence of novel cases of cancer and fatalities in the United States. The organization gathers statistics on population-based cancer incidence and outcomes by amalgamating data obtained from central cancer registries for incidence rates and the National Center for Health Statistics for mortality rates. As per their calculations, it is anticipated that there will be a total of 1,958,310 new cancer cases and 609,820 cancer-related fatalities in the United States by the year 2023 [2]. In the academic context, it can be observed that the incidence and mortality rates for various types of cancers, such as lung, colorectal, female breast, and prostate, have been declining in several western countries including the United States [3]. However, there has been an increase in these rates in certain developing nations with growing economies due to the adoption of unhealthy western lifestyles [3–5]. These unhealthy habits include smoking, physical inactivity, and the consumption of calorie-dense foods. As a result of these lifestyle changes, some of these countries have already surpassed western countries like the United States in terms of rates for lung and colon cancers [3].

The development of pathophysiological conditions like cancer is widely attributed to excessive cellular oxidative stress. Healthy cells utilize various mechanisms to maintain intracellular levels of reactive oxygen species (ROS) and overall redox homeostasis, thus preventing damage to DNA, proteins, and lipids [6]. The disturbance of the equilibrium between pro- and anti-oxidants, commonly referred to as oxidative stress, has been identified as a causative and pathophysiological factor in numerous chronic illnesses that are prominent causes of death [7, 8]. A growing body of research suggests that the consumption of specific nutrients such as vitamin C, vitamin E, and carotenoids (such as lycopene, beta-carotene, and lutein) may offer protection against oxidative stress [9–12]. Conversely, behaviors such as smoking and high iron intake can elevate the production of reactive oxygen and nitrogen species and hasten cellular damage related to oxidative stress [13–15]. The proposed measure, known as the oxidative balance score (OBS), has been utilized to assess the overall pro- and antioxidant exposure status in various chronic disease studies [16, 17]. Generally, dietary OBS is computed by combining pro- and antioxidant components that have been previously identified. Saturated fat, the proportion of n-3 to n-6 unsaturated fatty acids, total iron from the diet and supplements, and alcohol use are pro-oxidants. Total vitamin C, E, D, zinc, selenium, calcium, total beta-carotene, lycopene, alpha-carotene, lutein and zeaxanthin, cryptoxanthin, retinol, and gamma tocopherol were among the antioxidants. The body mass index, physical activity, and smoking are certain lifestyle characteristics that are also included in this index and are occasionally adjusted in some publications. In a particular study, it was determined that there exists a substantial negative relationship between OBS and colorectal adenoma when analyzed with community or endoscopic controls. This relationship was observed due to the exposure of pro- and anti-oxidants [18]. Despite the existing mechanisms regarding the positive effects of antioxidant balance in conditions such as malignancies, the results of studies are contradictory. Some studies indicate the positive and protective effects of OBS in cancer [19], while others have not mentioned significant results [20].

Overall, due to the existence of inconsistent findings, there is a need for a meta-analysis that will summarize all of the available studies in this field. So, the current meta-analysis was carried out to pool the association between oxidative balance score and cancer.

## Materials and methods

### Search strategy

This systematic review and meta-analysis was designed and implemented to assess the association of oxidative balance score and cancer morbidity in accordance with Preferred Reporting Items for Systematic Reviews and Meta-Analyses (PRISMA) guidelines [21].

We conducted a systematic review through which all related documents were searched in databases, including Web of Science, Embase, PubMed, Scopus, and Google Scholar until August 2023. The main routes for keyword combination extracted from ((“Oxidative Balance score” OR “OBS”) AND (“cancer” OR “Neoplasms” OR “tumor” OR “Malignancy”)) and all related terms. Also, to identify additional studies, the references cited within the relevant articles were reviewed. Two of the authors searched mentioned databases to identify eligible studies independently, and all articles obtained from a comprehensive search were managed by EndNote X9 software (Clarivate Analytics, PA, USA).

### Study selection

Studies were eligible for inclusion criteria in this review that assessed the relationship(s) of oxidative balance score with cancers in human with no limitation for the time of publications or researches, the language of papers, or gender. PECO was defined as a structured question describing the Population (adults with any medical malignancies), Exposure (oxidative balance score), Comparison (control group), and Outcome (incidence of cancer). Data from the searched papers were transferred to the Endnote software libraries. Duplicate records were excluded. To assess the eligibility and relevancy of included articles, in a three-step process, titles, abstracts, and full-text screening were conducted by two expert researchers independently.

Articles that had any of the following criteria were excluded from the meta-analysis: 1) trials; 2) studies which conducted in animals or labs; 3) other outcomes except cancer morbidity was reported such as mortality rate; 4) Studies that did not calculate and report the score related to oxidative balance; 5) review articles, editorials, letter to editors and case report studies.

### Quality assessment and data extraction strategy

The results of searches were evaluated by two of the authors based on mentioned the inclusion and exclusion criteria and extracted data from the studies independently. In extraction step, a checklist was used that included general, methodological and exposure and outcome related characteristics such as the first author, study country, study design, publication year, sample size, type of FFQ questionnaire, age and gender of participants, score of oxidative balance, exposure levels, type of cancer, detection methods and effect size. If a study had reported results for different cancer type (even both gender separately or in a specific gender), the reported effect sizes (EFs) were considered as a different study in meta-analysis. In some of the included studies, based on the stage of cancer progression, the participants were divided into two groups, non-advanced and advanced, and we reported the results in the same way. While, if there was no report on the stage of disease progression in the studies, we reported them as typical cases.

The quality of the included documents were assessed by using the Newcastle–Ottawa Quality Assessment scale [22]. Concerning risk of bias assessment, the Kappa statistic for the agreement between two researchers was 0.94. Probable discrepancy resolved under the supervision of the third expert opinion.

### Data analysis

The effect size for the association of OBS with cancers was considered Odds ratio (OR) with 95% confidence interval (CI), as same as, in cohort studies, the effect size was reported as hazard ratio (HR) with 95%CI.. The statistical heterogeneity between studies was evaluated by the Chi-square-based Q-test and the I^2^, and the result of the Q-test was considered to be statistically significant at *P* < 0.1. According to the value of I^2^, in case of severe heterogeneity (I^2^ >  ~ 75%), the pooled effect size was estimated conducting a random-effect meta-analysis model (with the Der-Simonian and Laird method). Also, for present the result of meta-analysis schematically, the forest plot was used. To identify the possible source of heterogeneity, subgroup analyses were performed according to the type of cancers, gender, case type, control type, and study design. Also, sensitivity analysis was conducted based on one out remove method (removing one article at a time and recalculating the pooled effects) to detect the potential outliers.

Publication bias was evaluated using Egger's test and funnel plots. Therefore, in order to find the source of publication bias, we performed the trim and fill test. All analyses were performed by STATA software vision 17 (StataCorp, College Station, TX, USA). A *P*-value < 0.05 was considered statistically significant.

## Results

### Literature search

**Fig. 1:**
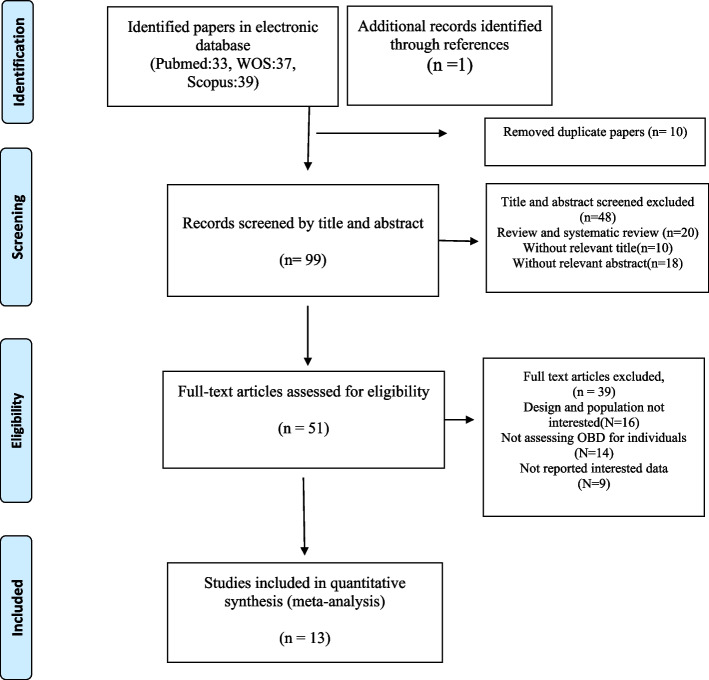
Flow diagram of the number of studies selected into the meta-analysis

In our initial search, 109 articles were identified. After exclusion of duplicate articles and those that did not meet the inclusion criteria, we identified 51 full-text articles of potentially relevant studies were identified. After full-text review, were excluded an additional 39 studies because their design and population were not interested or did not evaluate the association between OBS and cancers. Finally, by reviewing the full texts for cited references and then qualitative synthesis, fifteen observational studies were eligible for systematic review. A flow diagram of the study selection is shown in Fig. 1.

### Characteristics of the included studies

The characteristics of the included studies are provided in Tables 1 and 2. The total number of participants in these studies ranged from 173 [23] to 80,063 [24] subjects, with an age range between 47.6 [25] and 70.45 [20] years. In total, 266,737 participants (cohort studies = 249,485 and case–control studies = 17,252) were enrolled in the 15 studies included in the current systematic review [18–20, 23–31]. Six of the included studies were cohort [19, 20, 23, 24, 27, 29] and the rest were case–control studies. The follow-up periods ranged from 2 [25] to 26 [29] years in the cohort studies. Four studies included only men [20, 23, 27], and four publications involved women [19, 25, 29, 31]. The remaining articles were conducted on both genders. Diets in all studies were assessed using FFQ. Seven studies were used “FFQ 150 items” [25–27, 30, 31], three studies used “FFQ 152 items” [20, 24, 32], one study used “FFQ 98 items” [28], one study used “FFQ 110 items” [19], one study used “FFQ 127 items” [29],and one study used “FFQ 166 items” [23], and one study used “FFQ 106 items” [33]. Of the 15 included studies, one study was conducted in Europe [27], three in Asia [25, 32, 33], and the rest was conducted in USA. Eight of the studies related to colorectal cancer [18, 24, 26, 28–30, 32, 34], four prostate cancer [20, 23, 27, 34], and 3 other studies focused on breast cancer [19, 25, 31], and one study reported the gastric cancer [33].

**Table 1:** Characteristics and findings of included case–control studies

Author, Year	Design	Participants, n	Cancer type	Age	Case type	Control type	Gender	Method	Effect Size (OR)
Goodman 2008 [18]	Case–control	2305	Colorectal	Cases:59 Community controls: 59	Typical case	Population-based control	Both	FFQ 153-item + non dietary lifestyle pro-oxidant (smoking & alcohol & obesity) + non dietary lifestyle antioxidant (physical activity)	0.92 (0.88, 0.97)
Goodman 2010 [34]	Case–control	226	Colorectal	Cases:50 Community controls: 53	Typical case	Population-based control	Both	FFQ 153-item + non dietary lifestyle pro-oxidant (smoking & alcohol & obesity) + non dietary lifestyle antioxidant (physical activity)	0.34 (0.13, 0.88)
Goodman 2010 [34]	Case–control	323	prostate	Cases:54 Community controls: 52	Typical case	Population-based control	Male	FFQ 153-item + non dietary lifestyle pro-oxidant (smoking & alcohol & obesity) + non dietary lifestyle antioxidant (physical activity)	0.34 (0.14, 0.86)
Slattery 2012 [30]	Case–control	5311	Colon	Cases: 55.1 Control: 54.9	Typical case	Population-based control	Both	FFQ 150-item + non dietary lifestyle pro-oxidant (smoking & alcohol& obesity)	0.52 (0.41,0.66)
Slattery 2012 [30]	Case–control	1713	Rectal	Cases: 53.5 Control: 54	Typical case	Population-based control	Both	FFQ 150-item + non dietary lifestyle pro-oxidant (smoking & alcohol& obesity)	0.49 (0.35,0.70)
Dash 2013 [26]	Case–control	2289	Colorectal	CPRU case: 58 controls: 53 MAPI case: 58 controls: 55 MAPI case: 58 controls: 53	Typical case	Subcohort	Both	FFQ 150-item + non dietary lifestyle pro-oxidant (smoking & alcohol& obesity)	0.54 (0.43,0.69)
Dash 2013 [26]	Case–control	2289	Colorectal	CPRU case: 58 controls: 53 MAPI case: 58 controls: 55 MAPII case: 58 controls: 53	Non-advance	Subcohort	Both	FFQ 150-item + non dietary lifestyle pro-oxidant (smoking & alcohol& obesity)	0.60 (0.46,0.79)
Dash 2013 [26]	Case–control	2289	Colorectal	CPRU case: 58 controls: 53 MAPI case: 58 controls: 55 MAPII case: 58 controls: 53	Advance, stage3,4	Subcohort	Both	FFQ 150-item + non dietary lifestyle pro-oxidant (smoking & alcohol& obesity)	0.39 (0.26,0.58)
Kong 2014 [28]	Case–control	365	Colorectal	Cases: 56.9 Control: 55.9	Typical case	Population-based control	Both	FFQ 98-item + non dietary lifestyle pro-oxidant (smoking & alcohol & obesity) + non dietary lifestyle antioxidant (physical activity)	0.93 (0.88,0.99)
Slattery 2014 [31]	Case–control	2775	Breast	Non-hispanic White Case: 56. Control: 56.6 U.S.Hispanic or mexican Case: 52.7 Control: 52.3	Typical case	Population-based control	Female	FFQ 150-item + non dietary lifestyle pro-oxidant (alcohol)	0.74 (0.64,0.84)
Sohouli 2022 [25]	Case–control	520	Breast	Q1:49.2, Q2:50.4 Q3:47.6, Q4: 46.3	Typical case	Population-based control	Female	FFQ 150-item + non dietary lifestyle pro-oxidant (smoking & alcohol & obesity) + non dietary lifestyle antioxidant (physical activity)	0.51 (0.26,0.97)
Sohouli 2022 [25]	Case–control	520	Breast	Q1:49.2, Q2:50.4 Q3:47.6, Q4: 46.3	Premenopausal woman	Population-based control	Female	FFQ 150-item + non dietary lifestyle pro-oxidant (smoking & alcohol & obesity) + non dietary lifestyle antioxidant (physical activity)	0.34 (0.13, 0.88)
Sohouli 2022 [25]	Case–control	520	Breast	Q1:49.2, Q2:50.4 Q3:47.6, Q4: 46.3	Postmenopausal woman	Population-based control	Female	FFQ 150-item + non dietary lifestyle pro-oxidant (smoking & alcohol & obesity) + non dietary lifestyle antioxidant (physical activity)	0.86 (0.33, 2.12)
Kim 2022 [33]	Case–control	1212	Gastric	Case: 53.75 Control: 53.84	New diagnosed GC cases	Population-based control	Both	FFQ 106-item + non dietary lifestyle pro-oxidant (smoking & alcohol & obesity) + non dietary lifestyle antioxidant (physical activity)	0.49 (0.33,0.71)
Bentyaghoob 2023 [32]	Case–control	213	Colorectal	Case: 58.2 Control: 57.7	New diagnosed CRC cases	Other patients without neoplastic disease	Both	FFQ 150-item + non dietary lifestyle pro-oxidant (smoking & obesity) + non dietary lifestyle antioxidant (physical activity)	0.23 (0.07,0.72)

**Table 2:** Characteristics and findings of included cohort studies

Author, year	Design	Participants, N	Cancer type	Age	Case type	Control type	Follow up (years)	Gender	Female ratio	Method	Effect size (HR)
Agalliu 2011 [23]	Cohort	423	Prostate	62.2	Non-advance	Population-based control	Case: 4.3 Control: 7.7	Male	All men	FFQ 166-item + non dietary pro-oxidant lifestyle (smoking & alcohol)	1.02 (0.70,1.48)
Agalliu 2011 [23]	Cohort	173	Prostate	62.2	Advance, stage3,4	Population-based control	Case: 4.3 Control: 7.7	Male	All men	FFQ 166-item + non dietary pro-oxidant lifestyle (smoking & alcohol)	0.91 (0.54,1.51)
Geybels 2012 [27]	Cohort	2039	Prostate	62.8	Non-advance	Population-based control	17.3	Male	All men	FFQ 150-item + non dietary lifestyle pro-oxidant (smoking & alcohol & obesity) + non dietary lifestyle antioxidant (physical activity)	1.19 (0.99,1.43)
Geybels 2012 [27]	Cohort	1196	Prostate	62.8	Advance, stage3,4	Population-based control	17.3	Male	All men	FFQ 150-item + non dietary lifestyle pro-oxidant (smoking & alcohol & obesity) + non dietary lifestyle antioxidant (physical activity)	1.19 (0.96,1.47)
Lakkur 2014 [20]	Cohort	734	Prostate	70.45	Non-advance	Population-based control	9	Male	All men	FFQ 152-item + non dietary lifestyle pro-oxidant (smoking & alcohol & obesity) + non dietary lifestyle antioxidant (physical activity)	1.02 (1.02,1.04)
Lakkur 2014 [20]	Cohort	174	Prostate	70.45	Advance, stage3,4	Population-based control	9	Male	All men	FFQ 152-item + non dietary lifestyle pro-oxidant (smoking & alcohol & obesity) + non dietary lifestyle antioxidant (physical activity)	1.14 (0.87,1.50)
Dash 2015 [24]	Cohort	80,063	Colorectal	Men:70.2 Woman: 68.1	Typical case	Subcohort	18	Both	Not report	FFQ 152-item + non dietary lifestyle pro-oxidant (smoking & alcohol & obesity) + non dietary lifestyle antioxidant (physical activity)	0.59 (0.49, 0.70
Dash 2015 [24]	Cohort	33,354	Colorectal	Men:70.2	Typical case	Subcohort	18	Male	All men	FFQ 152-item + non dietary lifestyle pro-oxidant (smoking & alcohol & obesity) + non dietary lifestyle antioxidant (physical activity)	0.57 (0.43,0.75)
Dash 2015 [24]	Cohort	46,709	Colorectal	Woman: 68.1	Typical case	Subcohort	18	Female	All woman	FFQ 152-item + non dietary lifestyle pro-oxidant (smoking & alcohol & obesity) + non dietary lifestyle antioxidant (physical activity)	0.65 (0.50,0.83)
Mao 2021 [29]	Cohort	33,736	Colorectal	61.6	Non-advance	Subcohort	26	Female	All woman	WFFQ 127-item + non dietary lifestyle pro-oxidant (smoking & alcohol & obesity) + non dietary lifestyle antioxidant (physical activity)	0.66 (0.54,0.80)
Park 2021 [19]	Cohort	50,884	Brest	59	Non-advance	Subcohort	9	Female	All woman	FFQ 110-item + non dietary lifestyle pro-oxidant (smoking & alcohol & obesity) + non dietary lifestyle antioxidant (physical activity)	0.92 (0.81,1.03)

### Findings from the meta-analysis of oxidative balance score and cancer morbidity in case–control studies

**Fig. 2:**
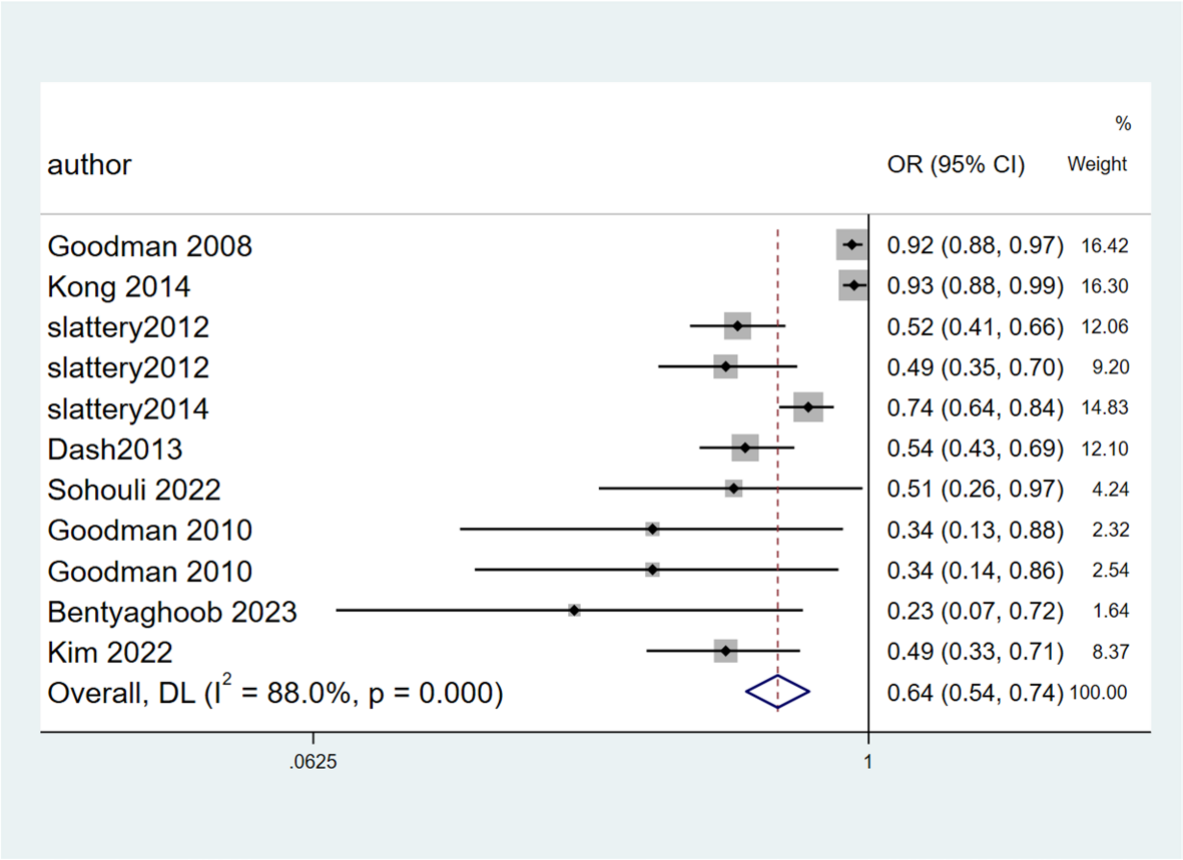
Forest plot of association between OBS and risk of cancers in case–control studies

Nine studies with 11 effect sizes and a total of 17,252 participants were included in this evaluation [18, 25, 26, 28, 30–34]. In the random- effect meta-analysis of case–control studies higher OBS was significantly decrease the odds of cancers (pooled OR: 0.64, 95% CI: 0.54, 0.74). However, there was evidence of significant heterogeneity between studies (*I*^*2*^ = 88%, Q test: 83.49; *P* < 0.01) (Fig. 2). No significant change was observed in the results by performing subgroup analysis based on the type of cancer and gender. In all investigated cancers, including colorectal (pooled OR: 0.66, 95% CI: 0.55, 0.79), breast (pooled OR: 0.71, 95% CI: 0.57, 0.89), prostate (OR: 0.34, 95% CI: 0.14, 0.86), and gastric (OR: 0.49, 95% CI: 0.33, 0.72), an inverse relationship between higher OBS and risk of cancer was found. Although the heterogeneity between studies decreased significantly in the breast cancer group and subsequently in the women group (*I*^*2*^ = 15.1.5%, Q test: 1.18; *P* = *0.278*) (Table 3).

**Table 3:** Subgroup-analysis of association between higher Oxidative balance score and risk of cancer

Higher oxidative balance score and odds of cancer in case-controls	Subgroups	Number of arms	OR (95% CI)	MODEL	Heterogeneity assessment		
					I^2^%	Q test	*P*-value
Gender	Both	8	0.64 (0.53.0.76)	Random	90.1	70.40	< 001
	Female	2	0.71 (0.56, 0.89)		15.10	1.18	0.278
Cancer type	Colorectal	7	0.66 (0.57.0.81)	Random	91.2	55.91	< 001
	Breast	2	0.71 (0.57, 0.89)		15.1	1.18	0.278
Higher oxidative balance score and risk of cancer in cohorts	Sub-group	Number of arms	HR (95% CI)	MODEL	Heterogeneity assessment		
					I^2^%	Q test	*P*-value
Stage of cancer	Typical case	6	0.86 ( 0.67,1.05)	Random	88.5	52.34	< 001
	Non-advance	3	1.05 (0.96, 1.14)		25.7	21.03	0.260
	Advance, stage 3,4	3	1.14 (0.97, 1.34)		0.0	1.12	0.640
Cancer type	prostate	3	1.15 (1.05,1.26)	Random	0.0	0.79	0.674
	colorectal	2	0.62 (0.54,0.71)		0.0	0.69	0.408
Gender	Male	4	1.05 (0.95,1.16)	Random	69.6	29.63	0.001
	Female	3	0.74 (0.58,1.06)		82.4	11.38	0.003

### Publication bias and sensitivity analyses

**Fig. 3:**
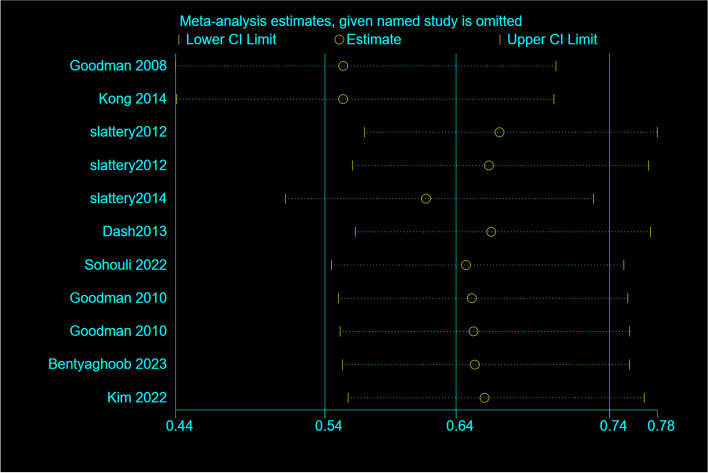
Sensitivity analysis: meta-analysis of random-effects estimates with studies omitted in case-controls

**Fig. 4:**
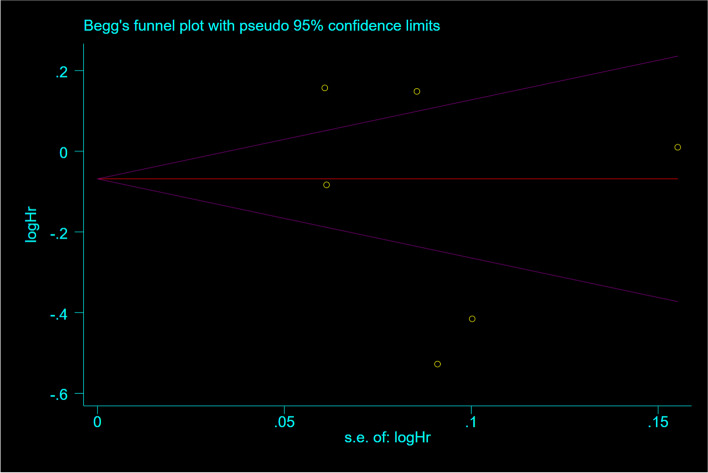
Funnel plot detailing publication bias in the case–control studies reporting the association between OBS and risk of cancers

Based on a random-effects model Sensitivity analyses revealed that excluding any single study from the analysis did not significantly change the pooled effect sizes (Fig. 3). Begg's regression test was assessed to evaluate publication bias, presented in (Fig. 4). Statistical Begg's test demonstrated no evidence of considerable publication bias for overall effect of OBS in the overall analysis (*p* = 0.52).

### Meta-regression analysis

Meta-regression showed that no significant association between OBS and female ratio (*p* = 0.77) and overall sample size (*p* = 0.75), age (*P* = 0.27) and follow-up duration (*P* = 0.71).

### Findings from the meta-analysis of higher OBS and cancer morbidity in cohort studies

**Fig. 5:**
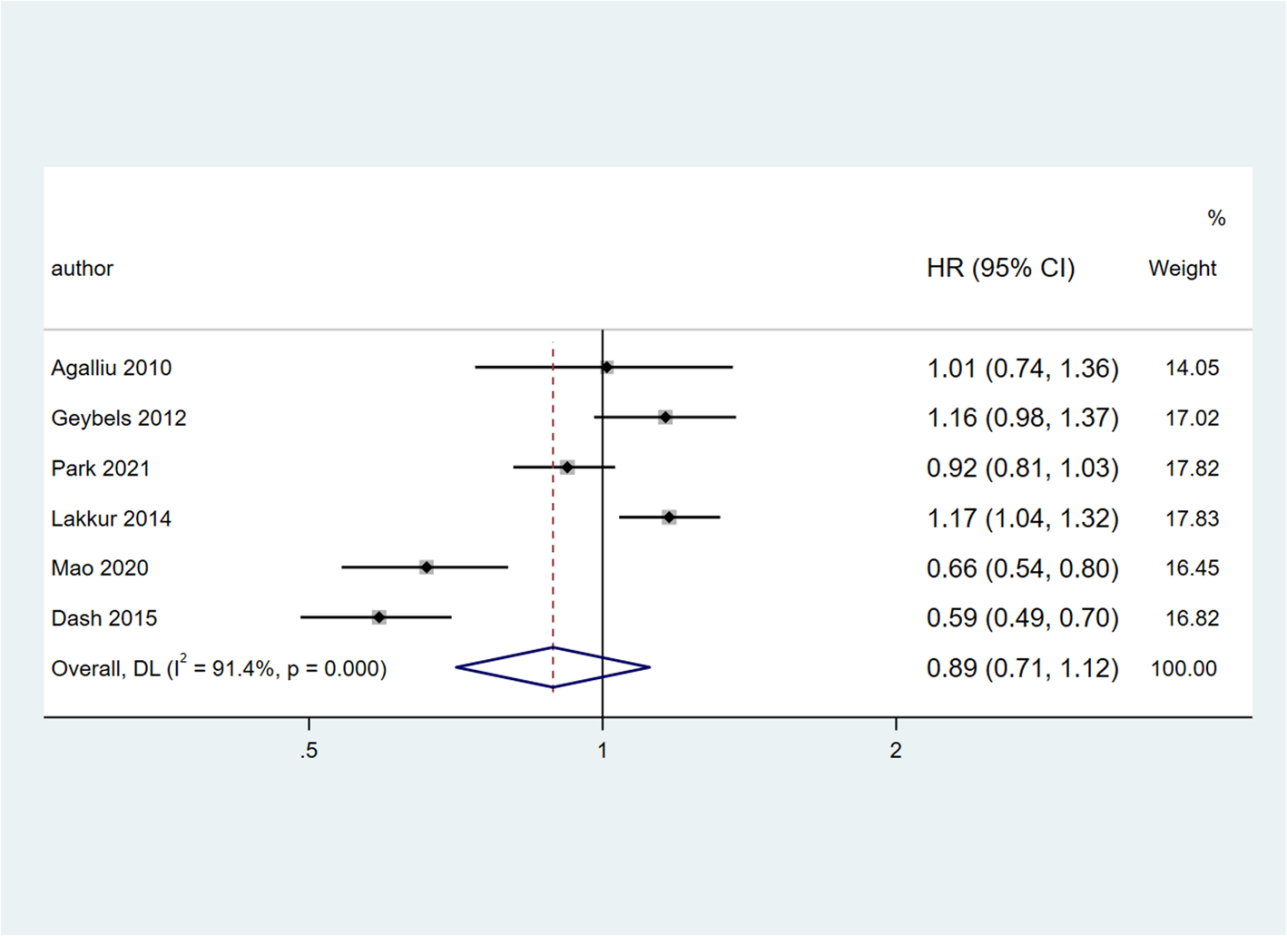
Forest plot of association between OBS and risk of cancers in cohort studies

Six studies with a total of 152,441 participants were included in the meta-analysis [19, 20, 23, 24, 27, 29]. The pooled HR of higher OBS and the risk of overall cancer was 0.89 (95%CI = 0.71, 1.12), which was not statistically significant. However, there was evidence of obvious heterogeneity between studies (*I*^*2*^ = 91.4%; Q test: 57.94; *P* < 001) (Fig. 5). Findings from subgroup analyses revealed that stage of cancer, cancer type and gender explained the between-study heterogeneity. Subgroup analysis based on cancer type showed a significant association between higher OBS and risk of prostate cancer (HR: 1.15 (95%CI = 1.05, 1.26), while in colorectal subgroup the results showed an inverse relationship between OBS and risk of cancer (HR: 0.62 (95%CI = 0.54, 0.71) but in breast cancer no significant relationship was observed (HR: 0.92 (95%CI = 0.82, 1.04)). Also, the degree of heterogeneity between studies reached the lowest value in both prostate cancer (*I*^*2*^ = 0.0%; Q test: 0.79; *P* = 0.674) and colorectal cancer groups (*I*^*2*^ = 0.0%; Q test: 0.69; *P* = 0.408).

Subgroup analysis based on gender shows a significant relationship between higher OBS and risk of cancer in female (HR: 0.74 (95%CI = 0.58, 0.96)) while this association in male gender was non-significant (HR: 1.05 (95%CI = 0.95, 1.16)). However, heterogeneity remained high in both groups. Moreover, subgroup analysis according to the stage of cancer, no significant results were found in any of the subgroups, while the heterogeneity reached negligible levels in the advance stage (3 & 4) group (Table 3).

### Sensitivity analyses and publication bias

**Fig. 6:**
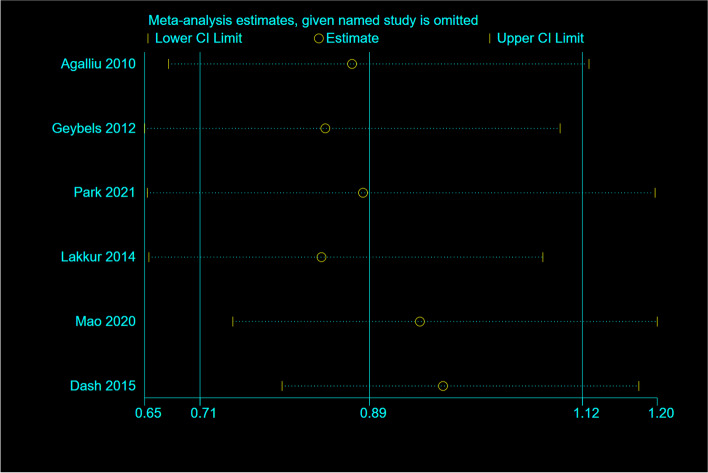
Sensitivity analysis: meta-analysis of random-effects estimates with studies omitted in cohorts

**Fig. 7:**
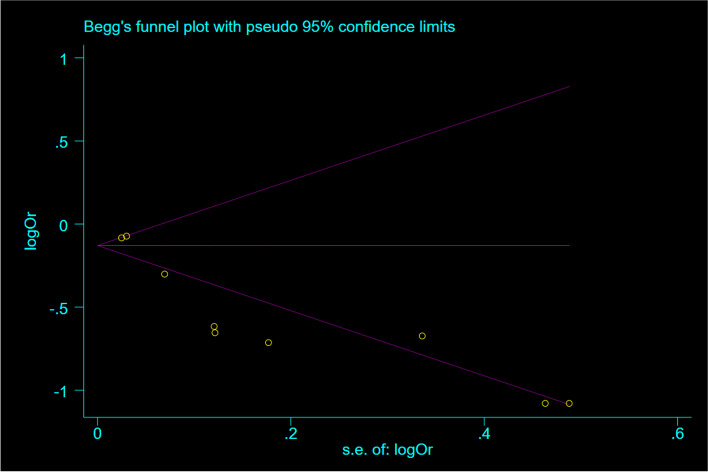
Funnel plot detailing publication bias in the cohort studies reporting the association between OBS and risk of cancers

Sensitivity analysis showed that, removing each study did not significantly change the overall effect (Fig. 6). According to the Begg’s test, no substantial publication bias was observed for the associations between OBS and risk of cancer (*P* = 0.46) (Fig. 7).

### Meta-regression analysis

A random-effect meta-regression analysis indicated no effect of female ratio, total sample size and follow up duration on pooled HR (*P* > 0.05).

### Quality assessment

The quality assessment of included studies was according to the modified Newcastle–Ottawa scale [35] for the type of study (cohort and case control) and were presented in Tables 4 and 5. The majority of the included studies achieved high scores as 7–10 points across domains.

**Table 4:** Newcastle–Ottawa assessment scale for cohort studies

Study	Selection				Compatibility		Outcome			Total
	**Representativeness of cases**	**Non-exposed cohort**	**Ascertainment of the exposure**	**Outcome of interest**	**On age**	**On other risk factors**	**Assessment of outcome**	**Follow up**	**Adequency of f/u**	
Agalliu 2011 [23]	*	*	*	*	*	*	*	*	*	9
Geybels 2012 [27]	*	*	*	*	*	*	*	*	*	9
Lakkur 2014 [20]	*	*	*	*	*	*	*	*	*	9
Dash 2015 [24]	*	*	*	*	*	*		*	*	8
Mao 2021 [29]	*	*	*	*	*	*	*	*	*	9
Park 2021 [19]	*	*	*	*	*	*		*	*	8

**Table 5:** Newcastle–Ottawa assessment scale for case–control studies

Study	Selection				Compatibility		Exposure			Total
	**Definition of cases**	**Representativeness of cases**	**Selection of controls**	**Definition of controls**	**On age**	**On other risk factors**	**Assessment of exposure**	**Same methods of ascertainment for cases and controls**	**Non response rate**	
Goodman 2008 [18]	*	*	*	*	*	*	*	*	*	9
Goodman 2010 [34]	*	*	*	*	*	*	*	*	-	8
Slattery 2012 [30]	*	*	*	*	*	*	*	*	*	9
Dash 2013 [26]	*	*	*	*	*	*	*	*	*	9
Slattery 2014 [31]	*	*	*	*	*	*	*	*	-	8
Kong 2014 [28]	*	*	*	*	*	*	*	*	*	9
Sohouli 2022 [25]	*	*	*	*	*	*	*	*	*	9
Kim 2022 [33]	*	*	*	*	*	*	*	*	*	9
Bentyaghoob 2023 [32]	*	*	*	*	*	*	*	*	*	9

## Discussion

The results of this meta-analysis showed that in case–control studies, higher OBS was associated negatively with odds of various cancers. Also, this relationship was stronger in breast cancer with negligible heterogeneity compared to colorectal and prostate cancer. On the other hand, in cohort studies, the overall relationship between OBS and all types of cancers was not statistically significant, although in the subgroup analysis there was a strong and positive relationship between OBS and prostate cancer, on the contrary, in other cancers such as breast, there was an inverse relationship, but statistically significant levels were not achieved. It is also noteworthy that subgroup analysis based on cancer stage did not significantly change the results and the relationship between OBS and cancer was insignificant in all stages. One of the possible reasons for the lack of discrepancy in the results between case–control and cohort studies is the design of them and actually the long-term follow-up period of the patients. Although the reverse causation should not be ignored. In fact, in case–control studies, because the participants are examined at one point in time, the priority and posterity of the exposure and the outcome are not clear, and it is possible that this score did not lead to cancer, but cancer. It has caused a change in diet and a change in this score. This issue can be the possible reason for non-significant results in cohort studies despite the presence of significant results in case–control studies.

The results of current meta-analysis in case–control studies are in line with existing theories about the protective effects of antioxidants against conditions with severe oxidative stress such as cancers. Based on our results, higher OBS is inversely associated with colorectal cancer risk. The consumption of pro-oxidants through diet, such as dietary iron, omega-6 fatty acids, and saturated fat, has been linked to an elevation in oxidative stress and DNA damage in the colon. This can be attributed to the increased production of free radicals in the colorectal region [22, 36–44]. The findings of a nested case–control study indicated a positive correlation between pre-diagnostic serum oxidized low density lipoprotein concentrations and the risk of colorectal cancer (CRC). These results were consistent with those obtained from another nested case–control study conducted within the European Prospective Investigation into Cancer and Nutrition cohort, which employed reactive oxygen metabolites and ferric reducing ability of plasma as indicators of oxidative stress [45, 46].

Numerous academic studies have investigated the correlation between an oxidative score, which comprises a comprehensive assessment of pro-oxidant and antioxidant exposure status, and the incidence of several types of cancer, such as colon/rectum, prostate, and lung [18, 47, 48]. The findings do not support the postulated theory that an advantageous equilibrium between pro- and anti-oxidant exposures shields against cancer formation and the results were not significant. On the other hand, in the subgroup analysis, the association between OBS and colorectal and breast cancer was consistent with the literature, but in the case of prostate, a positive and direct relationship was observed. Perhaps one of the reason for this discrepancy is the difference in scoring and evaluation methods of OBS. Agalliu et al. conducted a cohort study as part of the Canadian Study of Diet, Lifestyle, and Health. Their study revealed no correlation between OBS and the risk of prostate cancer as OBS quintiles increased. However, it is worth noting that the present study and three others utilized dissimilar categorizations for inclusion of components into the OBS, which could potentially explain discrepancies in results [23, 34, 49]. Geybels et al. study examined a more comprehensive list of OBS components than previous studies, which did not include α-carotene, zinc, flavonoids, or glucosinolates, all of which may act as antioxidants, and the results of this study, also, showed that there is no connection between OBS and prostate cancer [27]. Furthermore, the OBS utilized in the study by Lakkur and colleagues encompassed physical activity and body mass index as additional factors. It was observed that intense physical exertion contributed to a rise in reactive oxygen species production in the short term, whereas moderate physical activity facilitated upregulation of antioxidant gene expression via activation of Nrf2 [50]. The Agalliu and Goodman studies employed all polyunsaturated fats as a pro-oxidant, while Lakkur analyzed individual polyunsaturated fatty acids. Omega-3 fatty acids were classified as antioxidants due to their role in promoting the expression of antioxidant enzymes. In contrast, omega-6 fatty acids were identified as pro-oxidants, given that they act as precursors to pro-inflammatory eicosanoids [36, 51]. In contrast to the Agalliu study, Lakkur et al. conducted analyses that considered family history of prostate cancer. Recent researches have highlighted the potential role of oxidative stress as an environmental factor that may contribute to cancer pathogenesis, particularly in more severe forms of the disease [52, 53]. In prostate cancer, an augmented production of ROS as a result of metabolic processes of endogenous and exogenous compounds in prostate cells can lead to the formation of DNA adducts or direct DNA damage [54, 55]. Prostate cells must maintain a redox equilibrium between the production of ROS and the antioxidant defense mechanisms (e.g., vitamins C, E, β-carotene and selenium) that deactivate free radicals or couple them with glutathione increased ROS production or decreased antioxidant capacity may disrupt this balance and promote prostate cancer [56, 57]. It seems, the reason why the results of our study differed from the literature is that the number of included studies was small and the methods that each one developed and evaluated OBS were very different.

In the current systematic review and meta-analysis, we gathered all available evidence about the association between OBS and different cancer types. However, some potential limitation should be addressed. There was a considerable heterogeneity between the included studies which can be due to the small number of available studies and different methodology of studies. Also, due to the presence of reverse causation in case–control studies, it may not be possible to give a definite conclusion about the effect of OBS on cancer. It is suggested that more qualitative longitudinal studies be conducted in this field. One of the strong points of this study can be called the high quality of the studies included in this study, as well as the separate analysis of the studies according to the different effect sizes.

## Conclusion

The results of our study showed that OBS has an inverse relationship and a protective effect against cancers such as colorectal and breast in case–control and cohort studies. While the results of longitudinal studies indicated that there is a direct and positive relationship between prostate cancer and OBS. Due to the small number of studies on other types of cancers, we did not reach conclusive results, so it is recommended to conduct more studies in this regard.

## Data Availability

The datasets used and/or analyzed during the current study available from the corresponding author on reasonable request.
